# Mesenteric inflammatory myofibroblastic tumor mimicking acute appendicitis: A case report

**DOI:** 10.1016/j.amsu.2022.104456

**Published:** 2022-08-18

**Authors:** Saroj Kumar Yadav, Himal Bikram Bhattarai, Saurav Subedi, Anish Shrestha, Sangam Shah, Ayusha Subedi, Binita Kumari Yadav, Sajeev Joshi, Kavita Shah, Bidhan Bikram Shah

**Affiliations:** a Department of General Surgery, Koshi Hospital, Biratnagar, Nepal; b Gandaki Medical College, Pokhara, Nepal; c Tribhuvan University, Institute of Medicine, Maharajgunj, 44600, Nepal; d Manmohan Memorial and Community Hospital, Jhapa, Nepal; e Greencross Hospital, Biratnagar, Nepal; f Scheer Memorial Adventist Hospital, Banepa, Kavre, Nepal; g Nobel Medical College and Teaching Hospital, Biratnagar, Nepal; h Kathmandu Medical College and Teaching Hospital, Kathmandu, Nepal

**Keywords:** Inflammatory myofibroblastic tumor (IMT), Appendicitis, Mesentery

## Abstract

**Introduction:**

An Inflammatory myofibroblastic tumor, a neoplasm of intermediate biological potential, of the small bowel mesentery, is a rare tumor, most commonly reported in but not confined to the pediatric age group.

**Case presentation:**

This case report underlines a case of a (small bowel) mesentery IMT in an adult female presenting with recurrent symptoms similar to acute appendicitis.

**Discussion:**

It can present with symptoms similar to acute appendicitis necessitating a high index of suspicion for its prompt diagnosis. Treatment primarily includes surgical resection with recent advances in targeted therapy with tyrosine kinase inhibitors showing promising results.

**Conclusion:**

IMTs can present as clinical as well as histopathological mimickers of a variety of diseases especially in the abdomen. Prompt diagnosis requires both imaging and histopathological examination.

## Highlights

• Inflammatory myofibroblastic tumor is a neoplasm of intermediate biological potential of the small bowel mesentery.
• Prompt diagnosis requires both imaging and histopathological examination.
• Surgery still remains the main modality of treatment.

## 1 Introduction

Inflammatory myofibroblastic tumor (IMTs) comprises a heterogenous group of rare lesion that consists of predominantly myofibroblastic spindle cells intermixed with inflammatory cells [1]. Although IMTs can occur at any age, it is mainly described in children and young adult [2]. Because of obscure and inconclusive clinical presentation many times, IMTs need to be differentiated from other infectious, granulomatous, autoimmune, and malignant lesions. This case report underlines a case of a small bowel mesentery IMT in an adult female presenting with recurrent symptoms similar to acute appendicitis. This case has been described as per SCARE 2020 criteria [3].

## 2 Clinical presentation

A 50-year old female presented to the surgical OPD with a complaint of an intermittent abdominal pain located in the right lower quadrant since last 1 year. The pain was dull and aching in nature and had increased in severity for the last 3 days. She had a history of a similar episode 6 months back when she was diagnosed to have an appendicular lump which was managed conservatively in another center. This time, she presented with a similar type of abdominal pain to a different center where she was evaluated and diagnosed to have appendicular lump and was referred to our center for further management.

**Fig. 1:**
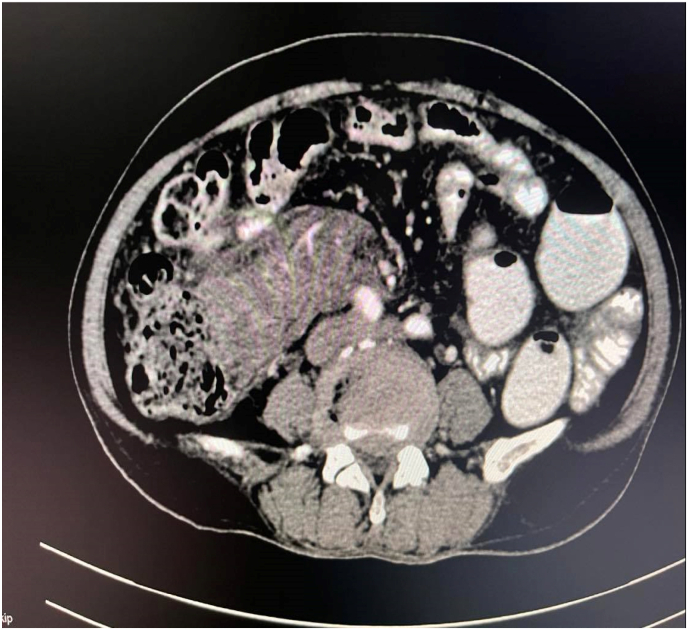
CECT abdomen and pelvis showing lump in mesenteric lump with traversing vessels.

The patient did not have complaints of weight loss or bleeding per rectum. Her appetite was normal. No other significant history was elicited. Her vitals were within normal limits and she had no pallor, icterus, edema, cyanosis, clubbing, or dehydration. On further evaluation, an ill-defined mildly tender mobile lump was palpable in the right lower quadrant of the abdomen. Her laboratory works were unremarkable except for a hemoglobin level of 9 gm %. A Contrast Enhanced Computed Tomography (CECT) scan was hence planned for further evaluation (see Fig. 1).

**Fig. 2:**
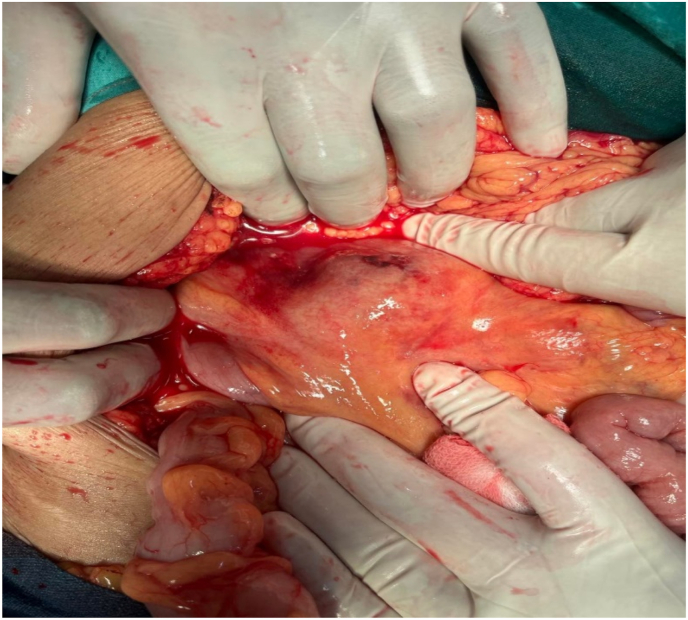
Intraoperative view of Lump.

The CT scan revealed a large heterogeneous mesenteric mass measuring 7.6*3.9 cm in the right lower abdomen, containing multiple enlarged lymph nodes with traversing mesenteric vessels abutting the medial aspect of the proximal ascending colon and caecum with surrounding mesenteric fat stranding densities and minimal fluid collection extending along the ascending colon up to the sub hepatic space (Fig. 2). There were a few irregular peripheral areas of hypodensity in the right liver lobe segment V and VI causing mild adjacent scalloping of the hepatic surface-one of which measures 2.6*1.5 cm. The spleen also had multiple irregular linear areas of hypodensity with the largest measuring about 1.9*0.8 cm in size.

With the above findings, the patient was planned for an elective laparotomy. Intra operatively an 8*4 cm firm, well defined lump was palpable in the right lower abdomen within the ileocolic mesentery and overlying traversing vessels were identified (Fig. 2). The mass was abutting the ascending colon and hepatic flexure and was surrounded by a jelly like material and friable tissue retroperitoneally extended to the Right kidney duodenum and ureter. Multiple enlarged lymph nodes were seen surrounding the lump. There was no intraperitoneal bleed or ascites.

Exploratory laparotomy with extended right hemicolectomy with ileotransverse anastomosis was done. Her postoperative course was uneventful and the patient was discharged satisfactorily on the fourth postoperative day.

**Fig. 3:**
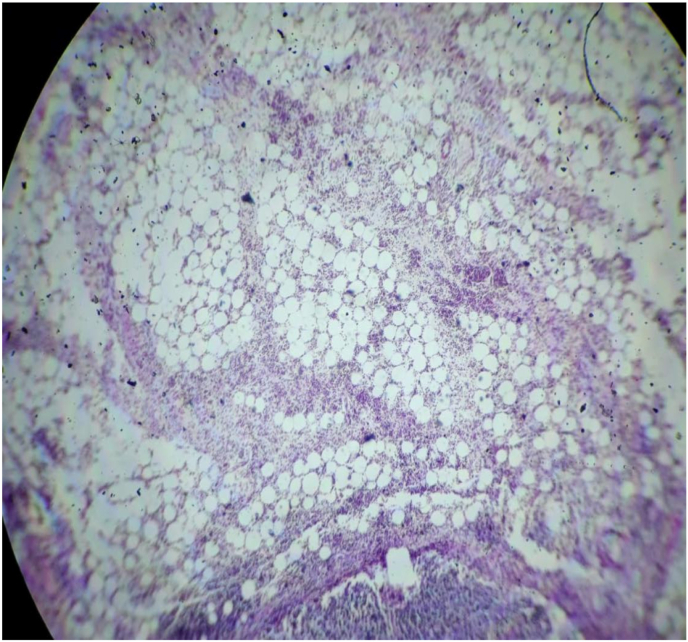
Histological slide showing mature looking adipocytes.

Finding of the specimen on gross examination showed a single well circumscribed solid firm mass within the mesocolon measuring 9*7*2 cm. Cut section of the specimen revealed a solid white homogenous area with central hemorrhage. The mass was 25 cm away from the colon and 5 cm away from the ileal resection margin. Three lymph nodes were identified during the procedure. Histopathological report of the mass showed the mass was hyper cellular with cells arranged in a compact fascicular and diffuse pattern in a myxoid background. Individual cells were spindle-shaped, round and elongated with abundant eosinophilic cytoplasm. Nuclei were oval to elongated in shape without atypical features and with occasional mitotic figures. There was a dense inflammatory cell infiltration predominantly consisting of plasma cells, eosinophil, neutrophils and lymphocytes. Mature looking adipocytes were also noted (Fig. 3). All the three lymph nodes examined were positive for tumor cells.

## 3 Discussion

Inflammatory myofibroblastic tumor is a rare mesenchymal tumor with unknown cause that encompasses a spectrum of myofibroblastic proliferation along with a varying amount of inflammatory infiltrates like lymphocyte, plasma cell and eosinophils [1]. IMTs occur in various age groups with a mean age of 9.7 years and a female preponderance [4]. Although the etiology and pathogenesis of IMTs remain unclear ALK gene rearrangements, viral infections (like Epstein-Barr virus, Human Immunodeficiency Virus, Human Herpesvirus-8), IgG4 Related Disease (more common in the head and neck region), trauma, chronic inflammation, and auto-immune diseases are some of the well-considered causes [5]. Despite this uncertainty of its cause, IMT is classified by the World Health Organization as a neoplasm of intermediate biologic potential under Fibroblastic/Myofibroblastic tumors because of its potential of local recurrences and distant metastasis [6]. Consistent with this, ALK rearrangements provide a clue to the neoplastic nature of the disease [7].

IMTs can involve almost every organ of the body. While the lungs are the most commonly involved site, IMTs have also been described in the mesentery, omentum, intestine, rectum, appendix, retroperitoneum, gastro-esophageal junction, mediastinum, liver, and abdominal wall. Among these, the most common extrapulmonary sites are the mesentery and the omentum as described in the above case presentation. Such extra-pulmonary IMTs are, however, more common among children rather than in adults [8]. Other sites where IMTs may arise include the pelvis, head and neck region, trunk, extremities, and skin [4,5]. Majority of patients with such IMTs present with an abdominal mass without other symptoms, however, a few IMTs may present with an acute abdomen as described above and some may also present with features of systemic inflammatory response syndrome [9]. Generally, the masses are nodular or globular and often with a myxoid background on gross examination. Sometimes features of necrosis and hemorrhage may be found. Histologically, three major variants of IMTs have been described which include spindle cell, mixed, and desmoid variants [10].

IMTs of abdomen have many differential diagnosis some of which include reactive/reparative lesions and mesenchymal tumors of the gastrointestinal tract including granulation tissues and nodular fasciitis in reactive processes, spindle cell sarcomas, peripheral nerve sheath tumors spindle cell melanomas, and sarcomatoid carcinomas, dedifferentiated liposarcoma, gastrointestinal stromal tumors, Hodgkin's lymphoma, inflammatory fibroid *polyp*, and other fibro-inflammatory processes (or pseudo neoplasms) and malignant tumors [11].

Despite the many speculations into the cause of IMTs, a complete resection of the tumor remains the mainstay of its treatment. Surgery is the most accepted treatment. If there is a biopsy-proven diagnosis of IMTs that excludes malignancy, medical treatment with no steroidal anti-inflammatory drugs in patients with peripheral hepatic IMTs could be prescribed [12]. Adjuvant chemotherapy regimens that have been reported are vincristine or vinorelbine with methotrexate, ifosfamide with carboplatin or doxorubicin and imatinib, are used for patients with incomplete tumor resection, positive margins and metastatic disease. Despite these reported adjuvant therapies, there have been no standardized treatment protocols due to lack of a definite efficacy. Thus, surgical treatment remains the cornerstone of management of various IMTs [13]. Despite this, incidence of local recurrence is reported to be 1 in every 4 cases ranging from 15 to 37% especially in the mesentery [9,14].

There have been recent advances in the treatment of IMTs with immunohistochemistry identifying ALK positive tumors. Such tumor have the potential of being treated with tyrosine kinase inhibitors targeting ALK which includes drugs like crizotinib and ceritinib [15].

## 4 Conclusion

IMTs can present as clinical as well as histopathological mimickers of a variety of diseases especially in the abdomen. Prompt diagnosis requires both imaging and histopathological examination. Despite recent advances in the pathophysiology of the disease, surgery still remains the main modality of treatment.

## Provenance and peer review

Not commissioned, externally peer-reviewed.

## Ethical approval

None.

## Please state any sources of funding for your research

No funding was received for the study.

## Author contribution

SS, AS, and SKY wrote the original manuscript, reviewed, and edited the original manuscript. SKY, HBB, SS, AS, SS, AS, BKY, SJ, KS, and BBS reviewed and edited the original manuscript.

## Please state any conflicts of interest

Authors have no conflict of interest to declare.

## Registration of research studies

1. Name of the registry: None
2. Unique Identifying number or registration ID: None
3. Hyperlink to your specific registration (must be publicly accessible and will be checked):

## Guarantor

Sangam Shah.

## Consent

Written informed consent was obtained from the patient for publication of this case report and accompanying images. A copy of the written consent is available for review by the Editor-in-Chief of this journal on request.
